# Social Conformity in Immersive Virtual Environments: The Impact of Agents’ Gaze Behavior

**DOI:** 10.3389/fpsyg.2020.02254

**Published:** 2020-09-08

**Authors:** Christos Kyrlitsias, Despina Michael-Grigoriou, Domna Banakou, Maria Christofi

**Affiliations:** ^1^GET Lab, Department of Multimedia and Graphic Arts, Cyprus University of Technology, Limassol, Cyprus; ^2^Research Centre on Interactive Media, Smart Systems and Emerging Technologies – RISE, Nicosia, Cyprus; ^3^Event Lab, Department of Clinical Psychology and Psychobiology, University of Barcelona, Barcelona, Spain

**Keywords:** virtual reality, agents, behavioral realism, social influence, conformity, social presence, eye contact

## Abstract

Immersive virtual reality (IVR) can induce an experience of “social presence” which can, in turn, increase social influence. Non-verbal behavior such as eye contact is an important component of human communication and, therefore, an important factor in creating social presence. This paper presents an experimental study that elaborates social influence through conformity with a group of virtual agents within an immersive virtual environment (IVE). Specifically, it investigates the impact of the agents’ gaze behavior on social presence and influence. An experiment based on the Asch (1951) paradigm using two levels of agents’ gaze behavior (Eye Contact condition vs. No-Eye Contact condition) was conducted. The results showed that participants conformed with the agents as they gave significantly more incorrect responses to the trials that the agents also gave an incorrect response, compared to those trials that the agents gave correct answers. However, no impact of the agents’ gaze behavior on conformity was observed, even if the participants in the Eye Contact condition reported a higher sense of social presence. In addition, self-reported measures showed a number of social effects that occurred only in the eye contact condition, indicating that the agents’ gaze behavior has an impact on participants’ experience.

## Introduction

In our daily lives, our decisions are greatly influenced by others. Our attitudes, our beliefs, and our behavior are influenced in a way that meets the demands of our social environment. This act of matching attitudes, beliefs, and behaviors to group norms, known as conformity, is one of the most powerful forms of social influence. This effect was initially studied by Jenness (1932), who asked the participants to estimate the number of beans in a bottle, individually. Then the participants were divided into small groups and were asked to discuss the task and to provide a common estimate, and finally, they were provided with the opportunity to revise their initial individual estimates. The results showed that the majority of the participants changed their initial estimation toward that of the group. Sherif (1935) conducted a series of experiments using the autokinetic effect, the illusion of movement in the absence of a reference point (spot of light in a dark room). When the participants were asked to individually estimate how far the light moved, their answers varied considerably. Yet, when they were asked to do the same in groups of three, stating their estimates out loud, Sherif found that their estimates converged. The most famous experimental approach of conformity, however, was the one carried out by Asch (1951, 1955, 1956). Asch conducted an experiment to investigate the extent to which social pressure from the majority can influence an individual and make him/her conform. Participants were placed in a room along with seven confederates and were asked to answer some simple line-length comparison tests. The confederates’ responses had been agreed in advance. The participant was led to believe that the other six attendees were also real participants and not part of the experiment’s scenario. The results demonstrated that the participants were affected by the pressure of the majority of others. Approximately one third of all estimates in the critical group were distorted in the direction of the majority.

One of the most common uses of Immersive Virtual Reality (IVR) technologies is the simulation of real or hypothetical scenarios (Slater and Sanchez-Vives, 2016) in entertainment, education (Stavroulia et al., 2018), training (Bombari et al., 2015), and research (Blascovich et al., 2002; Bombari et al., 2015), just to name a few. The use of Virtual Reality (VR) applications is expanding dramatically as IVR technologies are becoming more affordable and have the ability to provide a controlled, realistic, safe, and accessible experience to the user. Many of these scenarios include social interactions with agents, computer-generated representations of humans whose behaviors are determined by the computer (Blascovich et al., 2002; Von der Pütten et al., 2010) and who can play various social roles such as that of an audience (Nazligul et al., 2017) and teachers and students (Stavroulia et al., 2018). The social interaction with the agents plays a crucial role in the effectiveness of these applications, which requires that the agents behave as real people, so that the user reacts to them realistically (Bombari et al., 2015).

Recent studies have explored whether conformity can also be caused by virtual humans. The results of a study (Kraemer, 2013) replicated the Asch (1951) experiment within Second Life, a non-immersive virtual world that enables users to create virtual representations of themselves and interact with other users. The confederates in that study were avatars controlled by other users, and the participants were aware of that. The results showed that the participants were more likely to make the same choices as the confederates, compared to participants tested alone. A different study (Midden et al., 2015) compared the conformity with a group of humans, computers, and virtual agents displayed on computer screens. The results showed that conformity can be exerted by artificial majorities as participants conformed to the virtual agents’ group and the computer’s group only in a high-ambiguity task. The impact of the ambiguity of the task on conformity with non-humans was also addressed by Weger et al. (2015), who demonstrated that conformity was greater in more ambiguous tasks. Similar were the findings of a study reported by Hertz and Wiese (2016) that investigated social conformity with agents using three levels of human-likeness (computer, robot, and human). The results showed greater conformity in the high-ambiguity condition. Conversely, no effect of human-likeness on conformity was observed.

These studies, however, are limited to physical agents such as robots, and conversational agents on computer screens without the use of IVR technologies. IVR technologies have several advantages, which make them a very useful tool for reproducing social scenarios (Blascovich et al., 2002). One of the advantages of using IVR as a tool for psychological experiments is its ability to offer a high level of experimental control. This enables the researcher to conduct experiments which would be otherwise very difficult and inefficient.

An IVR system, thanks to its capability to provide a multisensory and interactive representation of a virtual environment, can induce to the user the illusion of being in this environment. This sense of “being there” is called presence (or place illusion) (Slater, 2009). Furthermore, IVR can induce an experience of “social presence” (or “co-presence”), the feeling of sensing another entity being present, which can in turn increase social influence (Oh et al., 2018). In this paper, we define social presence as the “sense of being together,” including the feeling that the “other” is a sentient human being (Oh et al., 2018). For example, a recent study (Bailey et al., 2019) showed that children in an IVR condition demonstrated social compliance to a greater extent than children in a non-immersive condition, suggesting that IVR may elicit differential cognitive and social responses compared to less immersive technologies.

Nonetheless, the exploration of social conformity within immersive virtual environments (IVEs) is still limited. The results of a study by Bailenson et al. (2008a, b) showed that the participants conformed to the virtual classmates who exhibited either positive (attentive and focused their gazes on the teacher) or negative (distracted and did not pay attention to the teacher) learning behaviors. Specifically, the participants’ learning abilities were affected by the virtual co-learners’ behavior in a virtual classroom.

A recent study (Kyrlitsias and Michael, 2016; Kyrlitsias and Michael-Grigoriou, 2018) replicated the Asch experiment in order to investigate conformity with virtual humans in IVEs using two experiments. The results of the first experiment showed that participants’ response time was affected by the virtual agents’ answers, indicating some level of social pressure, but the participants’ judgments were not affected. In a follow-up experiment, a similar procedure was used in order to investigate the effect of behavioral realism (gaze behavior) and agency (the extent to which the user believes that a virtual human is controlled by a real human rather than the computer) on conformity. The results showed that participants conformed to some extent to the virtual confederates, but no effects of agency and gaze behavior on conformity were observed. However, the level of conformity was very low, as only 4 of the 52 participants (7.69%) conformed to some extent. Nonetheless, this minimal conformity rate did not allow any clear conclusions to be drawn regarding the effect of gaze behavior and agency on conformity. Supplementary Table 1 summarizes the IVR experiments using the Asch paradigm, including the present study.

The primary aim of the current study was to investigate social conformity with a group of virtual agents within an IVE. To test that, we followed a procedure similar to the original Asch (1951) experiment, using the line-length comparison task. We designed an IVR version of Asch’s experiment, with four virtual agents as confederates. Based on our previous findings, and in order to achieve a higher conformity rate than in our previous study (Kyrlitsias and Michael-Grigoriou, 2018), we increased the task difficulty and also reduced the sense of anonymity of the participants. In order to increase the difficulty of the task, we limited the trial card projection duration, which is a common way of increasing the task difficulty in such experiments (Hertz and Wiese, 2016; Midden et al., 2015). Based on pilot tests, we balanced the task difficulty (projection duration and line-length differences), so that it was challenging enough, but the participants could still figure out the correct answer. In order to reduce the sense of anonymity, participants were asked to introduce themselves to the agents before the procedure began. Our prediction was that participants would conform to the virtual humans’ judgments by giving more incorrect answers to trials where the confederates gave a wrong response, than to the trials where the confederates answered correctly.

**FIGURE 1:**
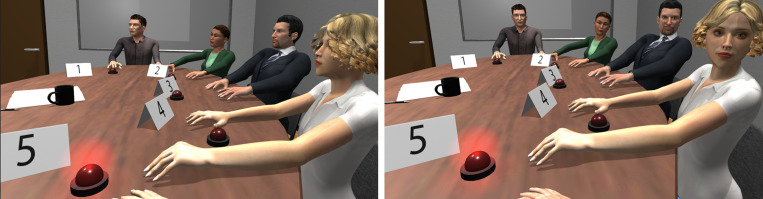
The virtual environment and the virtual agents as seen from the participant’s perspective in the NEC **(left)** and EC conditions **(right)** at a time in which the participant is required to provide an estimate.

Additionally, by manipulating the agents’ non-verbal behavior, and specifically the gaze behavior, we examined whether this factor has an impact on the level of conformity. Non-verbal behavior (such as eye contact, interpersonal distance, facial expressions, and gestures) is an important component of human communication (Bente et al., 2007) and, therefore, an important factor for the creation of social presence (Oh et al., 2018). In this study, we created two levels of gaze behavior for the agents, the Eye Contact (EC) condition and the No Eye Contact (NEC) condition. In the NEC condition, the agents had no gaze behavior, and therefore they did not make any eye contact with the participant or the other agents (Figure 1, left). In the EC condition, during the answering phases of the procedure, the agents turned their gaze toward the one who was responding at that moment, whether that was the participant or another agent (Figure 1, right). We presume that by adding an extra social cue, such as eye contact, a stronger sense of social presence will be induced to the participants, and, as social presence has an impact on social influence (Oh et al., 2018), we predicted that conformity in the EC condition would be greater than in the NEC condition.

This manipulation of the agent confederates’ gaze behavior is an example of the advantage of the use of IVR technologies for this kind of experiments. By using human confederates, it would be almost impossible to control their gaze behavior between the trials and the experimental sessions. Here the recruitment of virtual agents allowed us to have total control over the experimental protocol and study the impact of the confederates’ gaze behavior on participants’ conformity.

In addition, we measured the participants’ self-esteem (Rosenberg, 1965), a personality characteristic that is related to conformity (Gergen and Bauer, 1967), in order to understand how a person’s individual characteristics may affect social behavior with agents in IVEs. We also addressed the participants’ subjective evaluations regarding their experience in the virtual environment. The participants were asked to assess their sense of presence in the virtual environment and evaluate the agents’ behavioral realism. Moreover, we asked the participants to state how confident they felt about their answers and whether they were influenced by the responses of the virtual humans. Finally, using the head tracking provided by the VR headset, we recorded the participants’ gaze direction during their experience. Specifically, we recorded the duration that each participant looked toward the agents and the duration of the mutual gaze between the participant and the agents. This was done to examine the impact of social pressure and the agents’ behavior on the participants’ non-verbal behavior.

## Materials and Methods

### Experimental Design

Overall, 41 volunteers over 18 years of age participated in the study. One of them did not complete the experiment, while two participants were excluded as they were aware of the original Asch experiment on which the study was based, and which could have biased their responses. Therefore, in total, data collected from 38 participants, of whom 26 were female, were used in this study. This was a between-group design, and participants were randomly assigned to one of the two conditions, the EC group or the NEC group (Table 1). All participants signed a consent form which was a prerequisite for participation in the study.

**TABLE 1:** Distribution of participants over conditions and summarized pre-VR questionnaire measures.

	**Conditions**	
	**NEC**	**EC**
N (Males)	18 (6)	20 (6)
Mean ± S.E. Age	27.88 ± 1.845	24.4 ± 3.872
Median VR Experience (IQR)	2 (3)	3 (4)
Median 3D Experience (IQR)	2 (3)	2 (2)
Mean ± S.E. Self-Esteem	33.06 ± 0.979	29.05 ± 1.05

### Ethics Statement

All participants provided their written informed consent to participate in this study. Written informed consent was obtained from the individuals for the publication of any potentially identifiable images or data included in this article or the Supplementary Material.

### Technical Setup

The experiment was performed using a PC equipped with an NVidia GeForce GTX 1060 graphics card. The setup included the Oculus Rift (oculus.com/rift) head-mounted display (HMD) with 2160 × 1200 resolution (1080 × 1200 per eye), 110° field of view, and 90 Hz refresh rate for 3D immersive viewing, head rotational, and positional tracking, and providing spatialized audio. The application was created using the Unity (version 2018.2.1) game engine and the environment using Autodesk Maya and Adobe Photoshop. The virtual characters were designed and rigged using Autodesk Character Generator. For the lip synchronization feature, the SALSA plug-in for Unity was used.

### Virtual Agents

Four animated virtual agents were created for the experiment, two male and two female. Two body animation clips were created for each agent, an “idle” and an “answering” animation. The “idle” animation included breathing movements and was repeated for most of the time. The “answering” animation clip included some movement of the body and the hands and was playing each time the agents gave their answers. The above animation clips were slightly different for each agent. Also, to improve the realism, the agents performed blinking and lip movement animations using blend-shapes. The lip-movement animation was synchronized with the audio to simulate speaking. The audio clips used for the agents’ voice were pre-recorded.

An inverse kinematic technique directed by a scrip was used for the agents’ head movement and gaze manipulation. When the trials were projected, in both conditions the agents turned their heads toward the board. During the answering phase, in the EC condition all the agents turned their heads toward the one answering, including the participant (Figure 1, right), performing eye contact. The participants’ head position was tracked dynamically using the HMD’s positional tracking. During the answering phase in the NEC condition, the agents were looking straight ahead (Figure 1, left). An amount of randomness was applied to the delay and the speed of the agents’ head movement in order to make it look more natural and less robotic.

### Procedure

Upon their arrival at the laboratory, the participants received written information about the study and filled in the consent form. Then, they were asked to complete a pre-VR questionnaire that included demographic information as well as the Rosenberg self-esteem test.

After they were fitted with the virtual reality HMD and the necessary calibration was done, the virtual room with the 4 virtual agents (Figure 1) was presented and a familiarization period of 30 s began. Next, the instructions phase began, where pre-recorded instructions explaining the task and the process of the study were played back to the participant. During this phase, which lasted 2 min, the agents and the participant were asked to verbally introduce themselves by stating their first names, their age, and their occupation. This was done so that the participants could better understand the order and the way in which they would give their responses during the different trials, and to reduce the sense of the participants’ anonymity. Thirty seconds after the instruction phase was completed, the trial session began. Each trial was presented on the virtual boards for 5 s and then the agents and the participant gave their judgments in sequence. The participant was placed in the last (fifth) position and, therefore, gave his/her judgment after listening to the other four agents’ judgments. This procedure was repeated for all the 18 trials. Examples of different trial sessions for both experimental conditions are presented in Supplementary Video 1. More details about the trials are presented in the Trials section and Table 2. During this session, the participants’ answers to each trial (Trial Error) and the participants’ Response Time to each trial were automatically recorded by the software. Also, using the head tracking provided by the VR HMD, the Eye Contact Time, and the Mutual Gaze Time (in the EC group only) were recorded. More information about the recorded data can be found in the Measures section.

**TABLE 2:** Trials with the correct answers and the answers given by agents.

**Trial Number**	1*	2*	**3**	**4**	5*	**6**	**7**	**8**	**9**	10*	11*	**12**	**13**	14*	**15**	**16**	**17**	**18**
Correct Answer	A	B	A	C	A	C	C	B	A	A	B	A	C	A	C	C	B	A
Agents’ Answer	A	B	A	A	A	C	A	B	B	A	B	C	C	A	B	C	C	A

*Non-critical trials.

After the trials session, participants were asked to complete a post-VR questionnaire regarding their experience (Table 3). Finally, the participants were verbally asked whether they were familiar with the original Asch’s (1951) conformity experiment and they were debriefed.

**TABLE 3:** The questions of the post-VR questionnaire.

**Measures**	**Variable name**	**Question**
a. Presence	there	How do you assess your sense of presence in the virtual room where the trials were carried out?
	real	To what extent, during your experience, the virtual world has become the “reality” for you?
	lab*	During your experience, which sensation was stronger, the feeling that you were in the virtual room, or the feeling that you were in the laboratory where the study was being carried out?
b. Perceived Behavioral Realism	behave	The other participants in the study behaved like real people.
	move	The other participants were moving like real people.
	talk	The other participants spoke as real people.
	feel	I had the feeling that the other participants were real people.
c. Social Presence	sameRoom	I had the feeling that the other participants were with me in the same room.
	otherPerceived	I had the feeling that the other participants were aware of my presence.
	otherListen	I had the feeling that the other participants were listening to my answers.
	alone*	I had the feeling that I was alone in the room.
d. Response Confidence	correctAnswers	The answers I gave to the study were correct.
	difficult*	The tests were difficult.
	doubts*	I have doubts about the correctness of the answers I gave to the examination.
	confidentAnswers	I felt confident about my answers.
e. Self-Reported Conformity	myOpinion*	The answers I gave to the study were mainly based on my own opinion.
	otherOpinion	The answers given by the other participants in the study affected my own.

*Reverse interpretation.

### Trials

Overall, there were 18 line-length comparison trials. Each trial was presented for 5 s and had only one correct answer. The agents gave their answers in all trials unanimously.

Six of the trials (trials 1, 2, 5, 10, 11, and 14) were “non-critical,” and the agents gave the correct answer to all of them. The non-critical trials were used as training trials and were not considered in the analysis. The use of non-critical trials is a technique used in this kind of experiments (e.g., Asch, 1956; Hertz and Wiese, 2016), and their purpose was to avoid causing any confusion to the participants regarding the length comparison task and generate some trust toward the agents. This is the reason that the opening trials are non-critical.

The remaining 12 trials (3, 4, 6, 7, 8, 9, 12, 13, 15, 16, 17, and 18) were the “critical” trials. The agents gave the correct answer to the 6 critical trials (3, 6, 8, 13, 16, and 18) and a wrong answer to the other 6 (4, 7, 9, 12, 15, and 17). The correct answers to each trial as well as the answers given by the agents are summarized in Table 2.

The first 9 trials were identical with the other 9 in the same order (trial 1 was the same as trial 10, trial 2 as trial 11, and so on). In this way, each participant was asked to respond to each critical trial twice, once after the agents gave the correct answer and once after they unanimously gave a wrong answer. This was done in order to balance the task difficulty between the critical trials that the agents responded correctly to, and the critical trials where they gave a wrong answer. Participants were not aware of this manipulation.

### Measures

#### Pre-VR Questionnaire

Using a questionnaire that was given to the participants before their exposure to the virtual world, we recorded various data on demographic characteristics such as gender, age, intimacy with 3D environments and virtual reality, and self-esteem. These are summarized in Table 1.

Participants’ self-esteem was measured using the Greek version (Galanou et al., 2014) of the Rosenberg Self-Esteem Scale (Rosenberg, 1965). It includes a total of ten questions on a 1–4 consensus scale, and the score can range between 10 and 40. Higher scores are interpreted as higher self-esteem.

#### Post-VR Questionnaire

After their virtual exposure, participants were asked to complete a questionnaire (post-VR questionnaire) on their experience in the virtual world. All questions, which were evaluated on a 1–7 Likert scale, are presented in Table 3. The sense of Presence (Slater, 2009), the subjective sense of being in the virtual world, was recorded using three questions (Table 3, a) based on the Slater–Usoh–Steed (Slater et al., 1994) questionnaire. Four additional questions (Table 3, b) rated the realism of the agents’ behavior. Social presence was measured using four questions (Table 3, c) based on a questionnaire by Bailenson et al. (2003). With the use of 4 questions (Table 3, d), the participants stated the degree of confidence they felt about the answers they gave to the study, whereas two questions (Table 3, e) addressed whether they were influenced by the agents’ responses.

#### Trial Error

The responses given by the participants in each trial were recorded. Using these responses, a Conformity Error scale and a Non-Conformity Error scale were created. The Conformity Error represents the number of incorrect answers given by the participants in the trials that the agents gave the wrong answer. The Non-Conformity Error represents the wrong answers given in the critical trials where the agents gave correct answers. Additionally, a Conformity Index (CI) was constructed. The CI is a scale that describes the conformity magnitude in the agents’ responses. This scale was calculated from the difference of the Conformity Error and Non-Conformity Error (CI = Conformity Error – Non-Conformity Error) and describes the level of the participant’s conformity.

#### Response Time and Participants’ Gaze Behavior

Participants’ response time in each trial was recorded. Response time was the time distance between the moment the participants were called to respond and the moment they gave their answer in each trial.

The total duration that the participants were looking at the agents (we refer to this as Look-At Duration) was recorded. Due to the fact that the participants wore the VR headset that is not equipped with an eye tracker, a separate eye tracker could not be used. Thus, this measurement relied on the direction of the participant’s head, using the head tracking feature of the VR headset. Finally, the duration that the participants were looking at the agents when it was their turn to respond was recorded. At that time, in the Eye-Contact condition the agents also looked at the participants, which we refer to as Mutual Gaze Duration.

## Results

All results were obtained by analyzing data using the IBM SPSS Software v.24. The dataset generated for this study is provided in the Supplementary Data Sheet 1.

### Pre-VR Questionnaire

**FIGURE 2:**
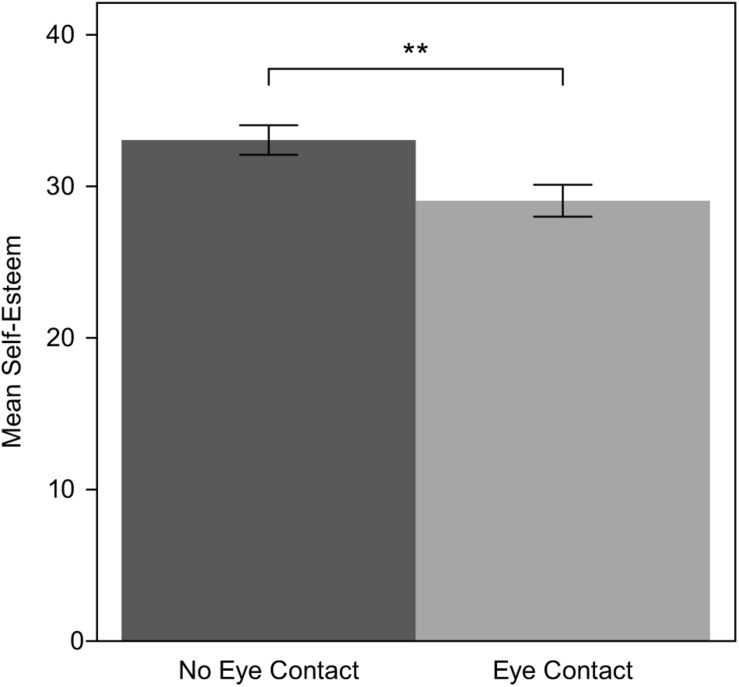
Means and standard errors of self-esteem in both experimental conditions.

In the NEC condition, the mean value of self-esteem was 33.06 while in the EC condition it was 29.05. The mean and the standard error of self-esteem score for each condition are shown in Figure 2. The mean value for both experimental conditions was 30.89, which is considered moderate self-esteem. An unexpected statistically significant difference was observed between the two conditions. A Mann–Whitney test showed that Self-Esteem was higher among participants in the NEC (*M* = 33.06, SD = 4.038) than those in the EC group (*M* = 29.5, SD = 4.696); *U* = 85.0, *p* = 0.009. This difference is taken into account in further analysis.

### Post-VR Questionnaire

In order to reduce the number of variables from the post-VR questionnaire (Table 3), a principal component analysis (PCA) was performed. A single factor emerged from each set of variables and the factor loadings in the scoring variables Presence, Perceived Behavioral Realism, Social Presence, Responses Confidence, and Self-Reported Conformity are shown in Table 4. The scoring coefficients are the coefficients of the equations describing the factor scores in terms of the linear combination of the original variables. The factor that emerged from the questions about presence (Table 3, a) is interpreted as “the feeling of ‘being’ in the virtual room.” The factor that emerged from the questions on agents’ behavioral realism (Table 3, b) is interpreted as “the extent to which the agents behaved like real people.” The factor that resulted from the social presence questions (Table 3, c) is interpreted as “the sense of being together with the agents.” The factor that emerged from the questions regarding participants’ responses confidence (Table 3, d) is interpreted as “the participants’ confidence in their responses.” Questions about self-reported conformity (Table 3, e) resulted in a single factor interpreted as “the statement that they were influenced by the agent’s answers.”

**TABLE 4:** Factor loadings and corresponding scoring coefficients for the factors resulted from principal component analysis.

**Variable**	**Factor loadings**	**Scoring coefficients**
**a. Presence**		
	F1	Presence
there	0.858	0.467
real	0.830	0.452
lab	0.641	0.349
**b. Behavioral Realism**		
	F1	Perceived Behavioral Realism
behave	0.895	0.320
move	0.821	0.293
talk	0.773	0.276
feel	0.852	0.305
**c. Presence**		
	F1	Social Presence
sameRoom	0.830	0.309
otherPerceived	0.866	0.322
otherListen	0.893	0.333
Alone	0.670	0.249
**d. Responses Confidence**		
	F1	Responses Confidence
correctAnswers	0.724	0.389
difficult	0.666	0.358
doubts	0.725	0.390
confidentAnswers	0.605	0.326
**e. Self-Reported Conformity**		
	F1	Self-Reported Conformity
myOpinion	0.924	0.541
otherOpinion	0.924	0.541

**FIGURE 3:**
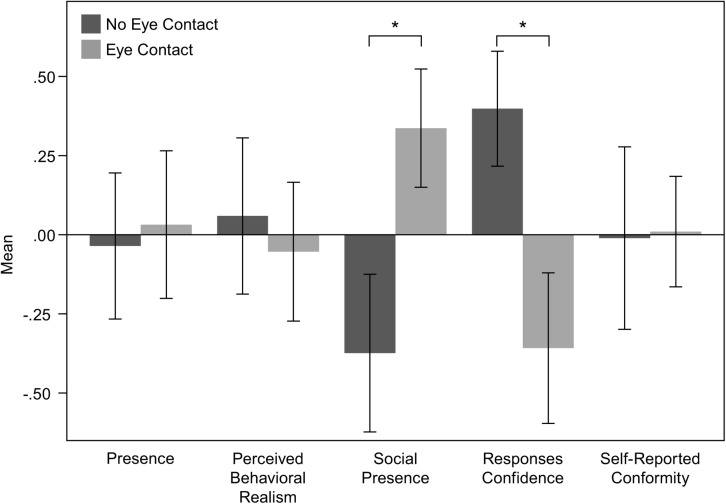
Means and standard errors of the variables resulted from factor analyses.

There was a statistically significant difference in Social Presence between the two experimental conditions. Specifically, participants in the EC condition reported higher sense of Social Presence (0.337 ± 0.187) than those in the NEC condition (−0.374 ± 0.249). An independent sample t-test showed that the above difference is significant; t(36) = −2.311, *p* = 0.027. An independent sample t-test showed a statistically significant difference in Response Confidence between the two conditions; *t*(36) = 2.485, *p* = 0.018. Participants in the EC condition (−0.358 ± 0.238) reported lower confidence about their responses to those in the NEC condition (0.398 ± 0.181). The means and standard errors of the derived variables are shown in Figure 3.

### Conformity

**FIGURE 4:**
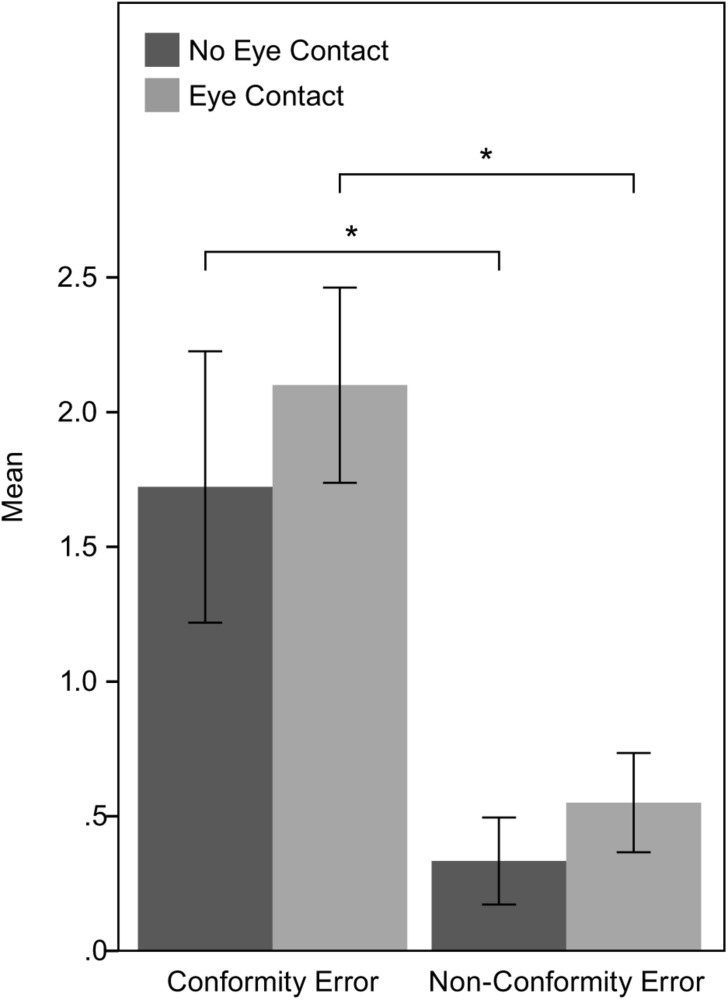
Means and standard errors of Conformity Error and Non-Conformity Error in both experimental conditions.

Initially, it was examined whether the participants’ responses were influenced by the agents’ responses in the critical trials. In order to do that, we compared the participants’ wrong answers in the trials where the agents replied correctly (Non-Conformity Error) with the participants’ wrong answers in the trials where the agents also replied wrongly (Conformity Error). A Wilcoxon signed-rank test showed a statistically significant difference between Conformity Error and Non-Conformity Error in both NEC condition; *Z* = −2.113, *p* = 0.035, and EC condition; *Z* = −3.001, *p* = 0.003. This result suggests that participants in both conditions conformed with the agents’ judgments, as they made wrong estimates more often in the trials where the agents gave a wrong answer, than in the trials where agents gave a correct answer. The means and standard errors of Conformity Error and Non-Conformity Error in the two experimental conditions are shown in Figure 4.

**FIGURE 5:**
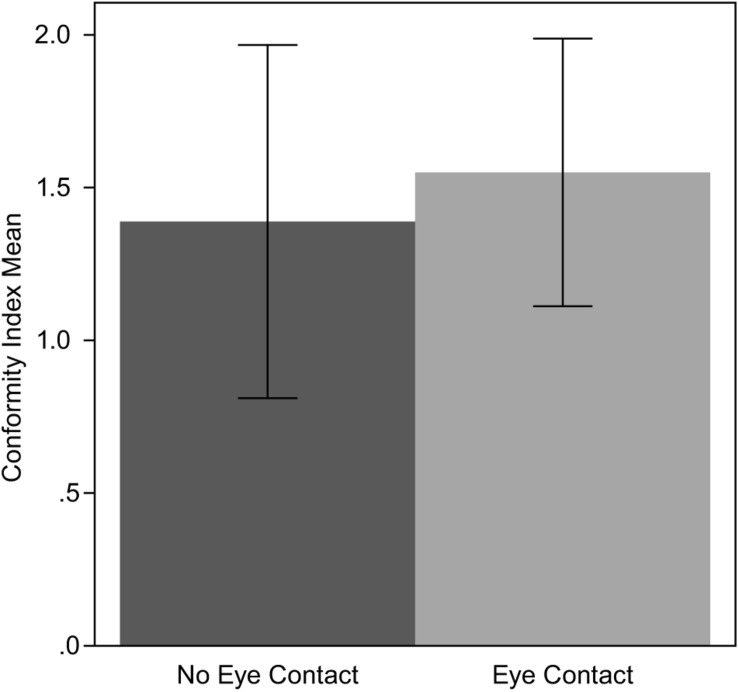
Means and standard errors of CI in both experimental conditions.

For the purposes of the analysis, we built the CI scale (described in Section “Trial Error”) to describe the level of the participants’ conformity. The median of the CI was 1, while the mean was 1.47. In the NEC condition, the mean was 1 while in the EC condition the mean was 1.5. An independent sample t-test indicated that this difference was not statistically significant; *p*(36) = −0.225, *p* = 0.823. This result suggests that the agents’ gaze behavior did not affect the conformity level. The mean and the standard error of CI for the two conditions are shown in Figure 5.

### Response Time and Participants’ Gaze Behavior

The response time of the participants’ answers in each of the critical trials was recorded, and the Mean Response Time was calculated. The mean response time of participants in the NEC group was 1.6 s, while in the EC Group it was 1.75 s. No significant difference in response time was observed between the two experimental groups; *t*(26) = −1.285, *p* = 0.210.

For the Look-At Duration, in the NEC condition the mean was 205.32 s while in the EC condition the mean was 192.8 s. This difference between the two conditions was not statistically significant (*t*(36) = 0.232, *p* = 0.818). However, this comparison between the two conditions could be influenced by the baseline difference in Self-Esteem (reported in Section “Pre-VR Questionnaire”), as the Look-At Duration was found correlated (reported in Section “Correlations”) with the participants’ Self-Esteem in the EC condition. For Mutual Gaze in the EC condition, the mean was 8.34 s. At the corresponding periods of the process, in the NEC condition, the participants looked at the agents altogether for an average of 8.26 s.

### Correlations

Participants’ Self-Esteem did not seem to be associated with the level of participant conformity. A Pearson product–moment correlation coefficient does not reveal any correlation between Self-Esteem and CI in any of the two experimental conditions. This result is important as it indicates that the difference in the baseline level of Self-Esteem that emerged between the two experimental conditions does not impact the results. Self-Esteem was only correlated with Look-At and Mutual Gaze duration in EC condition.

Finally, we looked at the correlations between the dependent variables in both experimental conditions and some interesting results have emerged. In both experimental groups, a correlation between conformity (CI) and Self-Reported conformity was found (NEC: *r* = 0.801, *n* = 18, *p* < 0.001; EC: *r* = 0.575, *n* = 20, *p* = 0.008), indicating that participants’ conformity was conscious. Another interesting result was that, in the EC condition, participants who stated that they were more confident about their responses responded more rapidly to the trials (*r* = −0.700, *n* = 15, *p* = 0.004). This correlation was not presented in the NEC condition. The correlation values and significance levels for the dependent variables in NEC and EC conditions are summarized in Tables 5, 6, respectively.

**TABLE 5:** Correlations between depended variables in the NEC condition.

	**1**	**2**	**3**	**4**	**5**	**6**	**7**	**8**	**9**
1. Conformity Index	-								
2. Self-Esteem	−0.132	-							
3. Social Presence	−0.247	−0.228	-						
4. Presence	−0.386	−0.241	0.433	-					
5. Self-Reported Conformity	0.801**	−0.246	−0.322	−0.308	-				
6. Perceived Behavioral Realism	−0.272	0.261	0.560*	−0.026	−0.362	-			
7. Response Confidence	−0.356	0.133	0.346	−0.076	−0.316	0.327	-		
8. Mean Response Time	−0.516	−0.426	0.007	0.403	−0.111	−0.524	−0.055	-	
9. Look-At Duration	0.072	−0.092	0.344	−0.044	−0.138	0.321	0.200	−0.288	-
10. Mutual Gaze Duration	0.020	−0.033	0.410	−0.049	−0.155	0.369	0.219	−0.283	0.918**

**Correlation is significant at the 0.01 level (2-tailed). *Correlation is significant at the 0.05 level (2-tailed).

**TABLE 6:** Correlations between dependent variables in the EC condition.

	**1**	**2**	**3**	**4**	**5**	**6**	**7**	**8**	**9**
1. Conformity Index									
2. Self-Esteem	0.306								
3. Social Presence	−0.120	−0.375							
4. Presence	0.014	−0.118	0.498*						
5. Self-Reported Conformity	0.575**	0.026	−0.160	0.117					
6. Perceived Behavioral Realism	0.156	−0.357	0.660**	0.503*	0.123				
7. Responses Confidence	−0.123	0.050	0.327	0.094	−0.256	0.293			
8. Mean Response Time	0.020	−0.282	−0.068	0.334	0.200	−0.182	−0.700**		
9. Look-At Duration	0.300	0.484*	0.007	0.189	0.232	−0.154	0.203	0.074	
10. Mutual Gaze Duration	0.281	0.467*	0.134	0.184	0.148	−0.075	0.277	−0.048	0.921**

**Correlation is significant at the 0.01 level (2-tailed). *Correlation is significant at the 0.05 level (2-tailed).

## Discussion

The first goal of this study was to investigate whether social conformity occurs with a group of virtual agents within an IVE. Our prediction was confirmed as participants’ judgments were significantly influenced by those of the agents. The participants gave significantly more incorrect responses to the trials where the agents gave a correct response, than the trials where the agents gave the correct response. This result has shown that within IVEs, conformity can be caused by the false judgments of a unanimous majority, even if the majority consists of artificial agents. In addition, the correlation between conformity and self-reported conformity, in both experimental conditions, indicates that the participants were consciously affected by the agents.

This finding is in line with the results of a previous study (Kyrlitsias and Michael-Grigoriou, 2018) where a similar result occurred. However, in the present study, the level of conformity is evidently higher, as only 7.69% of participants in Kyrlitsias and Michael-Grigoriou (2018) conformed with the agents, a percentage fairly small, in contrast to 63.16% of this study. We speculate that the increased level of conformity can be attributed to several differences between the two studies, which include the increased task difficulty, the sense of anonymity, and likely the VR equipment itself. With respect to the task difficulty, the literature has shown that the ambiguity of the task is a critical factor affecting the degree of conformity (Coleman et al., 1958). Specifically, participants tend to yield more easily to social pressure in a more difficult or ambiguous task than in an easier task. The difficulty of the task is also associated to the type of influence. In easy and obvious tasks, social conformity is attributed to normative influence (Deutsch and Gerard, 1955), as individuals change their judgment in order to match the group, but they keep their opinions private. On the other hand, with a difficult or unclear task, conformity can also be attributed to informational (Deutsch and Gerard, 1955) influence, as individuals change their judgment in order to be correct. In this study, we increased the difficulty of the task by projecting the stimulus for a limited duration (5 s). Another factor that may have affected the level of conformity is anonymity. Past studies have shown that the conformity rate is noticeably reduced when the responses are private (Gavish and Gerdes, 1998; Lea et al., 2001; Tsikerdekis, 2013). In VR, users are usually represented by virtual characters different to one’s own self-representation, which may give them the perception of some kind of anonymity. In this study, the participants were deliberately asked to verbally introduce themselves by stating their first names, their age, and their occupation, in order to decrease any sense of anonymity. Further studies need to investigate the impact of the user’s sense of anonymity on conformity in IVR. Finally, another factor that may have increased conformity is the level of immersion. In this study, we used an enhanced HMD (higher display resolution, better head tracking, etc.) than in our previous study (Kyrlitsias and Michael-Grigoriou, 2018). Past studies (e.g., Bailey et al., 2019) suggest that the level of immersion may affect social responses. Again, regarding conformity, further research is needed to confirm this speculation.

Our second prediction, that the inclusion of eye contact would increase the level of conformity with the agents, was not confirmed. This result replicates the outcome of another non-IVR research (Davey and Taylor, 1968), in which the authors attribute it to the fact that eye contact is only effective when combined with other social cues such as posture changes, gestures, and facial expressions. On the contrary, we confirmed our hypothesis that the eye contact manipulation can affect the sense of Social Presence. Specifically, participants in the EC condition stated significantly higher social presence than the participants in the NEC condition. However, the higher sense of social presence did not translate into a higher conformity level. Literature suggests that a higher sense of social presence leads to higher social influence (Oh et al., 2018), but it did not occur in this study on conformity, contrary to our prediction. An explanation of that is relevant to the type of conformity, which depends on the motives that led the participants to conform. Specifically, as mentioned above, the conformity in this case was informational, as the participants adopt the agents’ opinions in order to fulfill their desire to be correct, rather than to fit in, which is the case of normative conformity. An interpretation could be that informational conformity with agents does not depend on the humanization of the computer (which the case of social presence), but on the belief that the agents are reliable regardless to the extent on which they are perceived as social entities. In this case, the type of the task (line length comparison) may contribute, as computers are considered to be reliable in these types of tasks (Weger et al., 2015). This could be studied by testing the impact of social presence on conformity with agents in task that humans are considered as more reliable than computers (e.g., moral judgment). In that case, we believe that the sense social presence could affect the level of conformity.

Interestingly enough, the inclusion of eye contact as a social cue appears to influence the participants’ overall subjective experience. Participants expressed more doubts (lower responses confidence) about their responses when social pressure was exerted by agents who made eye contact. This finding suggests that although eye contact had some influence on participants’ decision-making process, it was probably not strong enough as there was no impact on their final responses. This finding can also be explained by the stronger sense of social presence that it is associated with social influence (Oh et al., 2018).

Nonetheless, no significant differences regarding the evaluation of the agents’ behavioral realism emerged between the two conditions. Participants in the EC condition, even though they stated that the agents felt more socially present, did not rate them as more realistic than the participants in the NEC condition. It is important to note here that the manipulation of the agents’ gaze behavior did not affect the perceived realism of the agents. Should the opposite have occurred, we would not be able to properly compare the two experimental conditions.

Some additional findings regarding the participants’ response times emerged between the two experimental conditions. Even though participants’ response time did not appear to be influenced by the eye contact manipulation, in the EC condition it was found to be associated with participants’ confidence. Specifically, participants in the EC condition who stated lower confidence in their responses took significantly longer to respond. This result is in line with the literature that suggests that post-decisional confidence is negatively correlated with choice latency (e.g., Zakay and Tuvia, 1998).

An unexpected setback of the study was the difference that arose in the reported Self-Esteem between the two experimental conditions. Participants in the NEC reported higher Self-Esteem than participants in the EC condition. In order to exclude the possibility that the results were biased due to these baseline differences, a correlation analysis was performed between self-esteem and each dependent variable under investigation. The analysis showed no correlations between Self-Esteem and any other dependent variable (e.g., conformity), except for the case of the two variables related to participants’ gaze behavior (Look-At Duration and Mutual Gaze Duration). Given this, the possibility that the results (e.g., for conformity) could be attributed to the difference in Self-Esteem can be rejected and safely attributed to the different condition. Regarding the measures related to the participants’ gaze behavior mentioned above (Look-At Duration and Mutual Gaze Duration) that were found correlated with Self-Esteem, it was shown that, in the EC condition, participants with higher self-esteem tended to turn their gaze more frequently toward the agents and performed more mutual gaze with the agents than did participants with lower self-esteem. This association is supported in the literature (Fugita et al., 1977; Vandromme et al., 2011).

The impact of participants’ Self-Esteem on their gaze behavior, observed in EC condition, consists of an interesting result that needs further investigation. Participants’ gaze behavior was not the focus of this study, and the data collected was not very accurate compared with data provided by an eye tracking HMDs available (e.g.,^1^). Hence, a more in-depth analysis of the participants’ gaze behavior was not possible. However, this study shows that the use of IVR and virtual agents can be ideal for this kind of experiments, thanks to its ability to provide a high level of experimental control between multiple experimental sessions.

This study confirms previous findings on the importance of designing artificial agents with realistic behavior toward the users in order to enhance one’s experience in IVEs. More interestingly, the findings suggest that the agents’ behavior may influence lower levels of conformity, by affecting the user’s decision-making. Further, the agents’ non-verbal behavior, such as eye contact as employed here, can have an impact on the sense of social presence, which has been shown to affect in turn the overall experience of the user.

This study showed that the creators or the moderators of IVR applications can use agents in order to influence and direct the users’ decision-making, through conformity. Social conformity is not limited to simple perceptual tasks, as in this experiment, but extends to other forms of behaviors and attitudes. The use of agents for indirect influence for the user could be used in various ways, such as directing the users of an IVR game in order enhance their game experience, or to influence them in a transaction, for example, in an immersive e-shop.

The findings of this study are very promising and highlight the need for further investigation in order to understand the factors that affect conformity with agents in IVEs. A factor that should be explored in a future study is whether agency (the extent to which the user believes that a virtual human is controlled by a real human rather than the computer) affects conformity. In this study, we showed that agents can elicit conformity; however, we do not know if the conformity will be greater with the use of avatars. Unfortunately, in this study we did not collect such data and we cannot know if the participants perceived the agents being controlled by a computer or by other real humans. Another important factor is the type of the task. In this study, we used a simple objective-perceptual task. The impact of the agents’ opinion on more social-objective tasks is also an interesting avenue to be explored in a future study.

## Data Availability Statement

All datasets generated for this study are included in the article/Supplementary Material.

## Ethics Statement

Ethical review and approval was not required for the study on human participants in accordance with the local legislation and institutional requirements. The patients/participants provided their written informed consent to participate in this study.

## Author Contributions

CK made substantial contribution in the conception and design of the study, in the data collection, in the analysis and interpretation of the data analysis, and in writing the article. DM-G substantially contributed in the conception and design of the study, in the interpretation of the data analysis, and in revising critically the manuscript, and she supervised and coordinated all the steps of the study. DB made substantial contribution in the design of the study, in data analysis, and in drafting and revising critically the manuscript. MC made substantial contribution in the acquisition of the data and in drafting and revising critically the article. All authors contributed to the article and approved the submitted version.

## Conflict of Interest

The authors declare that the research was conducted in the absence of any commercial or financial relationships that could be construed as a potential conflict of interest.

## Footnotes

1 vive.com/eu/product/vive-pro-eye
