# Supplementary material for: Social Conformity in Immersive Virtual Environments: The Impact of Agents’ Gaze Behavior
Source: Front Psychol. 2020 Sep 8;11:2254. doi: 10.3389/fpsyg.2020.02254 (PMC7506128; doi:10.3389/fpsyg.2020.02254)
Supplement: Supplementary file 1 [file Table_1.DOCX]

Supplementary Table 1: Summary of IVR Conformity Experiments including the present study.

| **Study** | **Equipment** | **Task** | **Type of Conformity** | **Projection Duration** | **Number of Subjects** | **Independent Variable(s)** | **Conformity observed** | **Conformity Error (%) *** | **Main Results** |
| --- | --- | --- | --- | --- | --- | --- | --- | --- | --- |
| Kyrlitsias & Michael, 2016; Kyrlitsias & Michael-Grigoriou, 2018 (study 1) | Oculus Rift DK1 | Line length comparison | Normative | Unlimited | 22 (13 in social pressure condition, 9 in no social pressure condition) | Social Pressure | No | 1.92% | No Conformity observed. |
| Kyrlitsias & Michael-Grigoriou, 2018 (study 2) | Oculus Rift DK2 | Line length comparison | Normative | Unlimited | 52 (6 or 7 participants in each condition) | Social Pressure, Agency, Eye Contact | Yes | 3.50% | Conformity observed.  No effect of Agency and Eye Contact on Conformity. |
| Present study | Oculus Rift (CV) | Line length comparison | Informational | 5 seconds | 38 (18 in NEC condition, 20 in EC condition) | Social Pressure (within subjects), Eye contact | Yes | 32.02% | Conformity Observed.  No effect of Eye Contact on Conformity. |
| *Wrong estimates on trials in which the agents gave a wrong answer. | | | | | | | | | |
